# Pancreaticoduodenal artery aneurysms and cerebral aneurysms in a patient of Sjögren’s syndrome: a surgical case report and literature review

**DOI:** 10.1186/s44215-026-00253-6

**Published:** 2026-03-29

**Authors:** Hiroto Yasumura, Koichiro Shimoishi, Sho Ijuin, Kenichi Arata, Yoshihiro Fukumoto, Goichi Yotsumoto, Yuki Ogata, Tomoyuki Matsuba, Yoshiharu Soga

**Affiliations:** 1 Department of Cardiovascular Surgery, Kagoshima City Hospital, Kagoshima, Japan; 2 Department of Gastroenterology, Kagoshima City Hospital, Kagoshima, Japan; 3 Department of Cardiovascular Surgery Graduate School of Medical and Dental Sciences, Kagoshima University, Kagoshima, Japan

**Keywords:** Pancreaticoduodeal artery aneurysm, Median arcuate ligament syndrome, Cerebral aneurysm, Sjögren's syndrome, Bypass

## Abstract

**Introduction:**

Pancreaticoduodenal artery aneurysms (PDAAs) are associated with celiac axis stenosis and occlusion. PDAAs carry a risk of sudden rupture and therefore require prompt treatment. Sjögren’s syndrome (SS), a systemic autoimmune disease, has recently been reported in association with various arterial aneurysms. To our knowledge, no previous report has described concurrent PDAAs and cerebral aneurysms (CAs) associated with SS. Here, we present a rare case of PDAAs and CAs associated with SS.

**Case presentation:**

A 69-year-old Japanese woman with a history of chronic hepatitis was referred to our hospital. She had a 20-year history of dry mouth, a one-month history of taste disturbance, and joint pain. Laboratory tests revealed she was SS. Abdominal ultrasonography performed for chronic hepatitis incidentally revealed aneurysms around the pancreas. Contrast-enhanced computed tomography (CECT) demonstrated multiple PDAAs, including two anterior superior PDAAs, three anterior inferior PDAAs, one inferior PDAA, and two dorsal pancreatic artery aneurysms. The origin of the celiac axis was occluded. CECT also revealed three CAs: an anterior communicating artery (AcomA) aneurysm with a bleb, a right middle cerebral artery aneurysm, and a right M1 bifurcation aneurysm.

We planned a three-stage treatment strategy. First, an aorto-hepatic bypass using a reversed great saphenous vein graft was performed. The graft was proximally anastomosed to the abdominal aorta 15 mm above the inferior mesenteric artery, routed through the mesocolon near the ligament of Treitz, and passed between the stomach and pancreas. It was then distally anastomosed to the common hepatic artery. The gastroduodenal artery (GDA) was ligated to prevent competitive flow between the GDA and the graft. The postoperative course was uneventful, and the patient was discharged on postoperative day 9. Twenty days later, coil embolization of the PDAAs was performed as the second treatment. Fifty days thereafter, stent-assisted coil embolization of the AcomA aneurysm was performed as the third treatment. Twelve months after the aorto-hepatic bypass, the graft remained patent, and the sizes of the remaining CAs were unchanged.

**Conclusions:**

Our case highlights the potential for systemic aneurysm formation in patients with SS and demonstrates that staged multidisciplinary treatment can be safely and effectively performed.

## Introduction

Pancreaticoduodenal artery (PDA) aneurysms (PDAAs) are rare, accounting for approximately 2% of all visceral arterial aneurysms [1]. They are often associated with celiac axis stenosis or occlusion, which may result from atherosclerosis, median arcuate ligament (MAL) syndrome (MALS), or tumor compression. Increased superior mesenteric artery (SMA) blood flow secondary to celiac axis stenosis or occlusion enhances retrograde flow through the pancreaticoduodenal arcade, thereby contributing to the formation of PDAAs [2]. PDAAs carry a high risk of sudden rupture and require immediate treatment regardless of size (Strength of Recommendation: 1, Quality of Evidence: B) [3]. One major therapeutic option for PDAAs due to celiac axis occlusion is aorto-hepatic bypass, followed by staged trans-arterial embolization (TAE) of the aneurysms [4].

Sjögren’s syndrome (SS), a systemic autoimmune disease, has recently been reported in association with various arterial aneurysms, including cerebral aneurysms (CAs) [5, 6], visceral artery aneurysms [7, 8], spinal artery aneurysms [9] and aortic aneurysms [10, 11], and coronary artery disease [12]. However, concurrent PDAAs and CAs in patients with SS have not been previously reported. Here, we report the first case of concurrent PDAAs and CAs associated with SS and discuss a multidisciplinary treatment strategy.

## Case presentation

A 69-year-old Japanese woman (height, 155.2 cm; weight, 68.2 kg) with a history of chronic hepatitis was referred to our hospital. She had experienced dry mouth for 20 years and presented with taste disturbance for one month and joint pain. She had been using artificial saliva for a long time. She had a family history of cerebrovascular disease but no history of autoimmune disease. She had never smoked. Physical examination revealed no other abnormal findings.

Laboratory tests showed elevated aspartate aminotransferase (82 U/L; normal range: ≦30 U/L), elevated alanine aminotransferase (107 U/L; normal range: ≦23 U/L), elevated γ-glutamyl transpeptidase (68 U/L ; normal range: ≦32 U/L), elevated C-reactive protein (0.25 mg/dL ; normal range: ≦0.14 mg/dL), elevated rheumatoid factor II (22.3 IU/mL; normal range: ≦15 IU/mL), elevated antinuclear antibody titer (1:640 ; normal range: <1:40), and elevated anti–SS A antibody (209 U/mL; normal range: ≦9.9 U/mL). Based on these findings, she was diagnosed with SS.

**Fig. 1:**
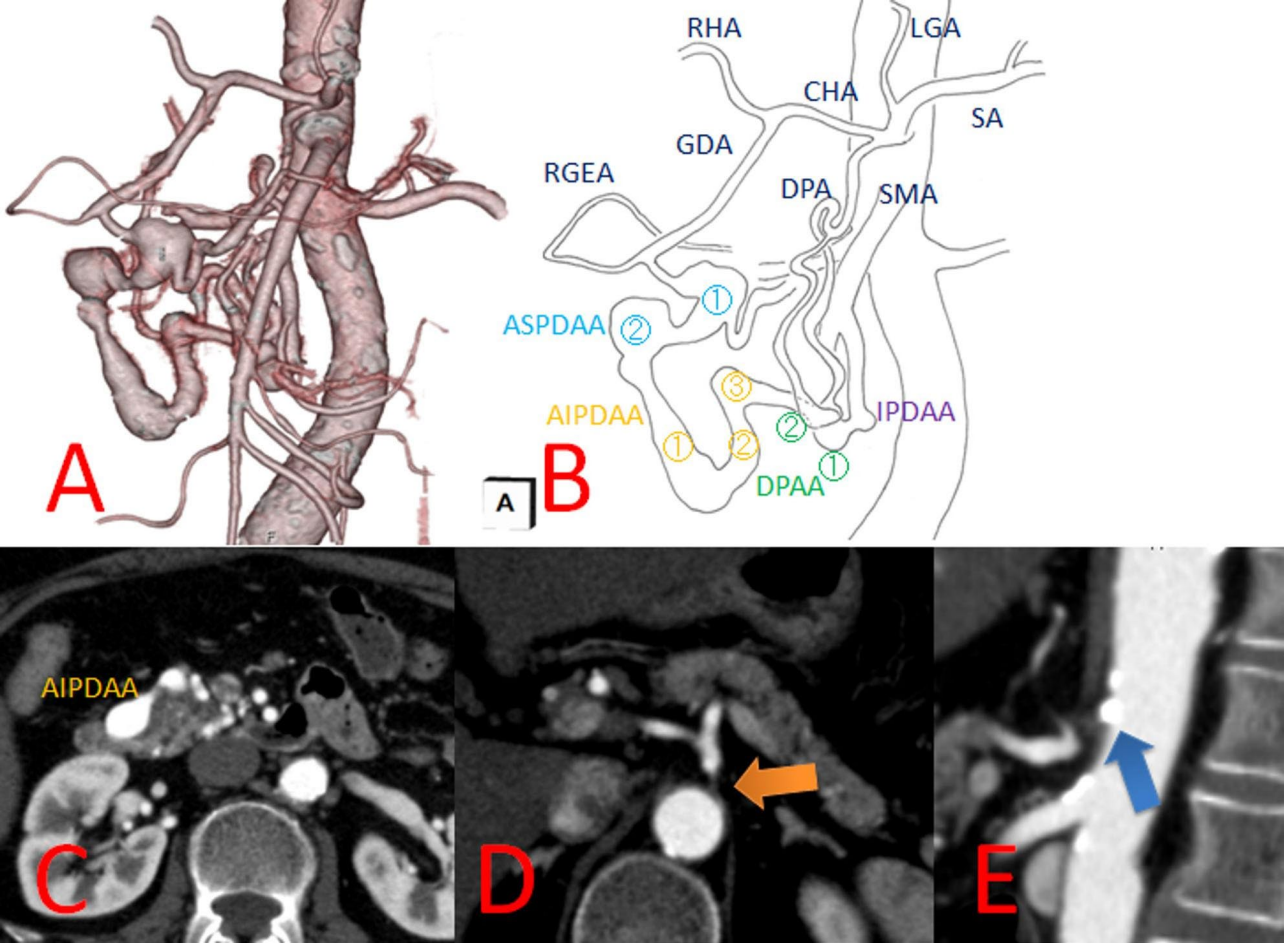
Preoperative contrast-enhanced 3D CT findings of visceral artery aneurysms. **A** Three-dimensional (3D) contrast-enhanced computed tomography (CT) showing pancreaticoduodenal artery aneurysms (PDAAs) and dorsal pancreatic artery aneurysms (DPAAs). **B** Schematic diagram of the 3D contrast-enhanced CT. **C** Contrast-enhanced CT revealed two DPAAs and six PDAAs, including two anterior superior PDAAs (ASPDAAs), three anterior inferior PDAAs (AIPDAAs), and one inferior PDAO (IPDAA). **D** The origin of the celiac axis was occluded (orange arrow). No post-stenotic dilation was observed. **E** Calcification at the origin of the celiac axis (blue arrow)

**Fig. 2:**
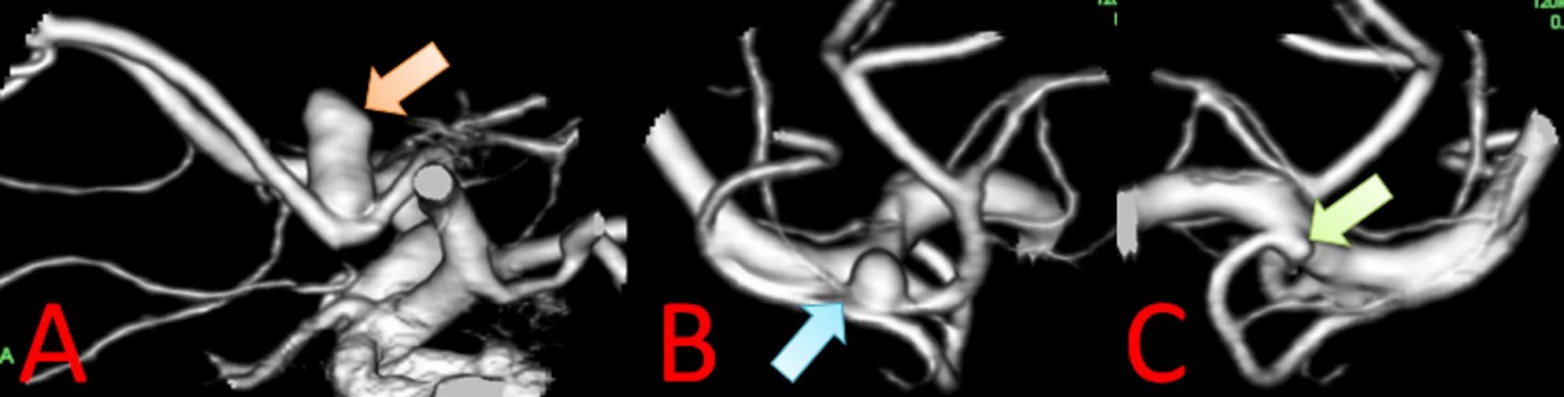
Preoperative contrast-enhanced 3D CT findings of cerebral aneurysms. **A** An anterior communicating artery (Acom) aneurysm (10 mm, orange arrow) with a bleb. **B** A right middle cerebral artery aneurysm (3 mm, blue arrow). **C** A right M1 bifurcation aneurysm (1.5 mm, green arrow)

Abdominal ultrasonography performed to evaluate liver dysfunction revealed fatty liver and pancreatic artery aneurysms. Liver biopsy findings suggested that the etiology of the chronic hepatitis was metabolic dysfunction–associated steatohepatitis (MASH). Contrast-enhanced computed tomography (CECT) revealed two dorsal pancreatic artery aneurysms (DPAAs), each measuring 6 mm, and six PDAAs (Figs. 1 A–C). These included two anterior superior PDAAs (ASPDAAs) measuring 12 mm and 14 mm, three anterior inferior PDAAs (AIPDAAs) measuring 12 mm, 8 mm, and 9 mm, and one inferior PDAA (IPDAA) measuring 8 mm. The origin of the celiac axis was occluded. There was no post-stenotic dilatation of the celiac axis (Fig. 1 D), and calcification was observed at its origin (Fig. 1E). Aortography also demonstrated occluded origin of the celiac axis. Moreover, CECT revealed three CAs: an anterior communicating artery (AComA) aneurysm measuring 10 mm in diameter with a bleb (Fig. 2A), a right middle cerebral artery aneurysm measuring 3 mm (Fig. 2B), and a right M1 bifurcation aneurysm measuring 1.5 mm (Fig. 2C).

**Fig. 3:**
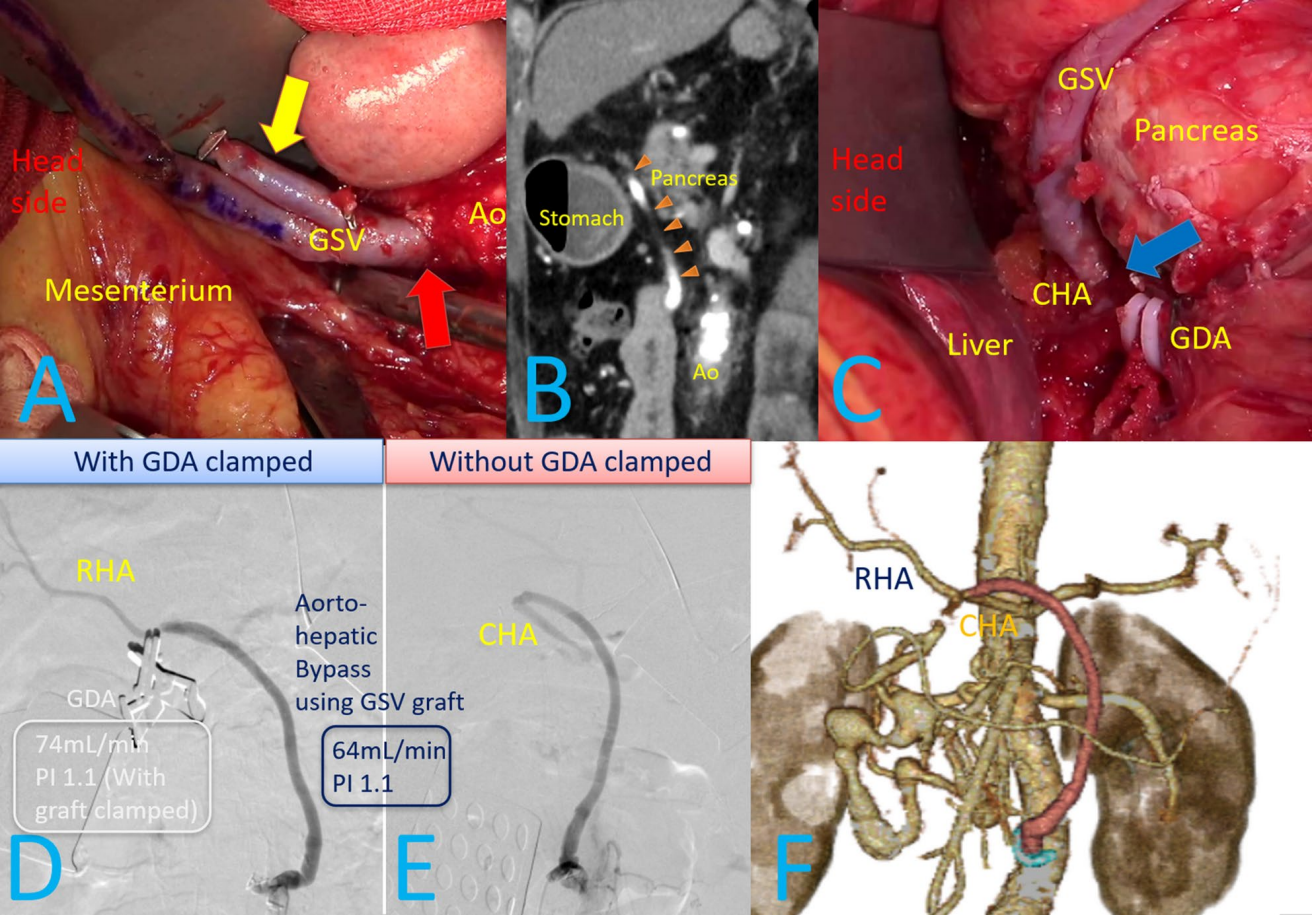
Intraoperative and postoperative findings. **A** A reversed great saphenous vein (GSV) graft (red arrow) anastomosed to the abdominal aorta, 15 mm above the inferior mesenteric artery, using an end-to-side parachute technique with 6-0 polypropylene. Yellow arrow is a branch of the GSV, which was preserved and used for intraoperative angiography. **B** To achieve the shortest route, the graft (orange arrowhead) was passed from the abdominal aorta to the common hepatic artery (CHA) through the mesocolon near the ligament of Treitz and between the stomach and pancreas. **C** The graft was anastomosed to the CHA (blue arrow) with an end-to-side parachute technique using 7-0 polypropylene. After angiography, the gastroduodenal artery (GDA) was ligated to prevent competitive flow between the GDA and the graft. **D** Angiography from the graft with the GDA clamped showed flow through the graft, right hepatic artery (RHA), and CHA. **E** Angiography from the graft without the GDA clamped showed flow only through the graft and CHA, with to-and-fro flow in the CHA. **F** Postoperative contrast-enhanced 3D CT demonstrated a patent aorto-hepatic bypass

We planned three staged treatments, including an aorto-hepatic bypass and TAE for the PDAAs and CAs. Although the AComA aneurysm was suitable for surgical clipping, the patient declined this option. As the first-stage treatment, two months after the diagnosis of PDAAs, an aorto-hepatic bypass using the great saphenous vein (GSV) was performed under general anesthesia. The GSV was reversed and anastomosed end-to-side to the abdominal aorta approximately 15 mm above the inferior mesenteric artery using a parachute technique with 6 − 0 polypropylene sutures (Fig. 3A). The graft was routed through the mesocolon near the ligament of Treitz and passed between the stomach and pancreas (Fig. 3B). The venous valves were incised using a Lemaitre^®^ valvulotome (LeMaitre Vascular Inc., Massachusetts, USA), and the graft was anastomosed end-to-side to the common hepatic artery (CHA) using a parachute technique with 7 − 0 polypropylene sutures (Fig. 3C). The length of the grafted GSV was 16 cm.

With the gastroduodenal artery (GDA) clamped, the graft flow was 64 mL/min, and the pulsatility index (PI) was 1.1 (Fig. 3D). In contrast, GDA flow with the graft clamped was 74 mL/min, with a PI of 1.1. Intraoperative angiography from a branch of the graft (Fig. 3A) without GDA clamped showed only the graft and CHA flow with a to-and-fro pattern at the CHA (Fig. 3E). However, angiography with GDA clamping demonstrated flow in the graft, right hepatic artery, and CHA (Fig. 3D). Therefore, the GDA was ligated to avoid flow competition between the GDA and the GSV graft (Fig. 3C). The postoperative course was uneventful, and CECT confirmed graft patency (Fig. 3F). Aspirin and apixaban were initiated to prevent bypass graft occlusion, and the patient was discharged on postoperative day 9.

**Fig. 4:**
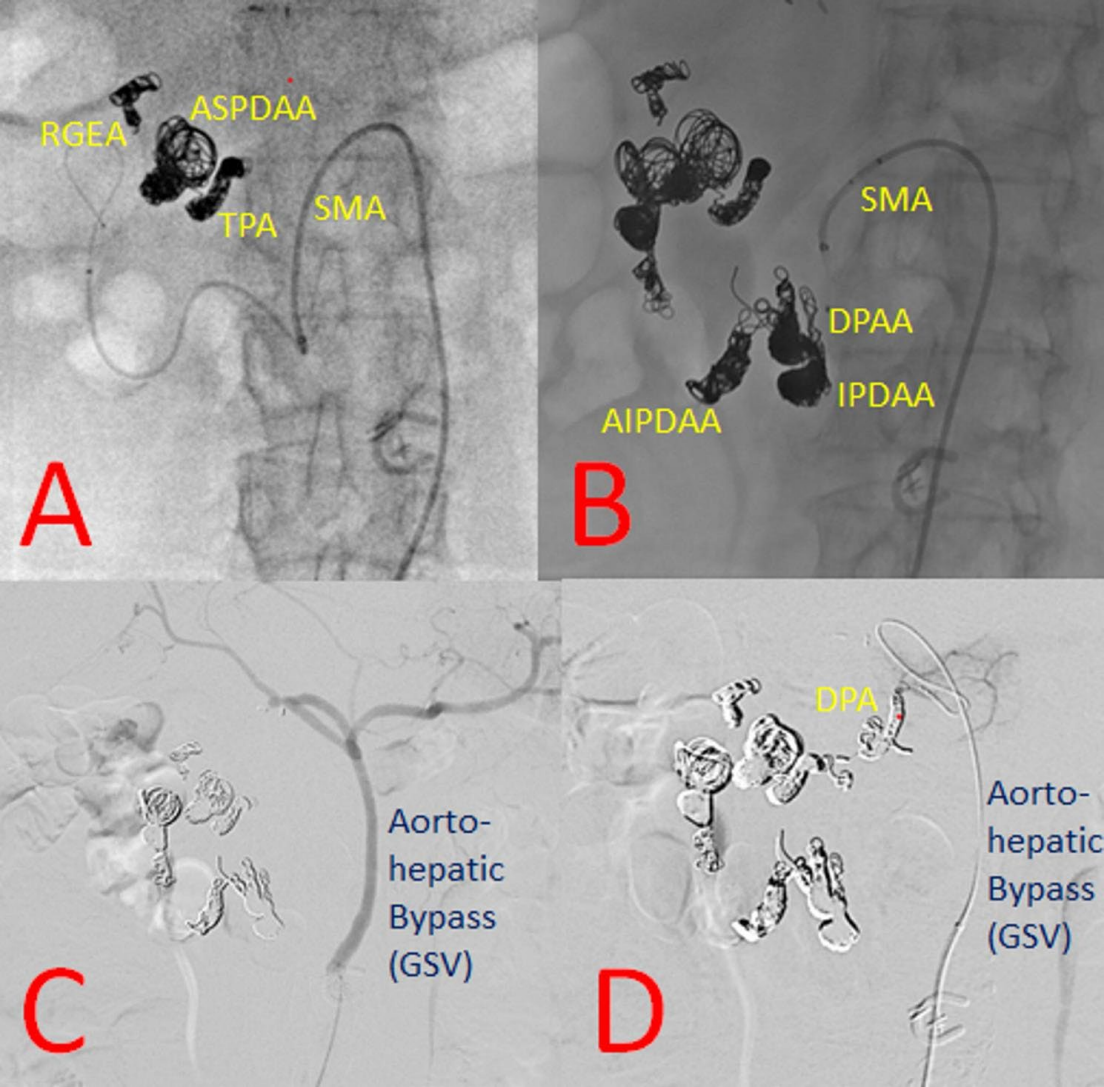
Trans-arterial embolization findings. **A** After superior mesenteric artery (SMA) catheterization, embolization was first performed at the origin of the right gastroepiploic artery (RGEA) and the anterior superior pancreaticoduodenal artery aneurysms (ASPDAAs) with a DELTAFILL (Johnson and Johnson, New Jersey, U.S.A.) and AZUR Soft 3Ds (TERUMO, Tokyo, Japan) , followed by embolization of the transverse pancreatic artery (TPA) with AZUR Soft 3Ds and C-STOPPERs (PIOLAX MEDICAL DEVICES, Yokohama, Japan). **B** Embolization of an additional ASPDAA was then performed with a MICURUSFRAME S (Johnson and Johnson, New Jersey, U.S.A.) and C-STOPPERs, followed by embolization at the origin of the anterior inferior PDAA (AIPDAA) and the inferior PDAA (IPDAA) with MICURUSFRAME Ss, DELTAFILLs, C-STOPPERs, Tornados (COOK MEDICAL, Indiana, U.S.A.), AZUR CX35s (TERUMO, Tokyo, Japan), and EMBOLDs (Boston Scientific, MA, U.S.A.). **C** Angiography from the aorto-hepatic bypass. **D** Embolization of another dorsal pancreatic artery (DPA) aneurysm was performed via the aorto-hepatic bypass with AZUR Soft 3Ds

Twenty days after the aorto-hepatic bypass, TAE was performed as the second-stage treatment. Following SMA catheterization, embolization was first performed at the origin of the right gastroepiploic artery and the ASPDA (Fig. 4 A). Second, the transverse pancreatic artery was embolized. Third, embolization of the ASPDAA was performed. Fourth, embolization at the roots of the anterior inferior PDA and posterior inferior PDA was carried out (Fig. 4 B). Because all dorsal pancreatic arteries (DPAs) were difficult to embolize via the SMA, angiography via the aorto-hepatic bypass was performed (Fig. 4 C), followed by embolization of the DPAs through the bypass (Fig. 4 D). The patient did not develop ischemic pancreatitis and was discharged on postoperative day 2.

Fifty days later, stent-assisted coiling (SAC) of the AComA aneurysm was performed under general anesthesia. SS was managed without steroid therapy because both the PDAAs and AComA aneurysm were successfully treated without complications. Twelve months after the aorto-hepatic bypass, the graft remained patent, and the sizes of the remaining CAs were unchanged.

## Discussion and conclusion

Regarding the etiology of true PDAAs and duodenal artery aneurysms, Corey [2] reported that 23 of 35 patients (65.7%) had calcific atherosclerosis at the celiac axis origin, and 11 patients (31.4%) had MALS at the celiac axis origin. Other causes of celiac axis stenosis include isolated celiac dissection [13]. The etiology of PDAAs can be diagnosed with CECT. In typical MALS, the MAL compresses the celiac axis, usually showing a characteristic superior indentation a few millimeters from the vessel origin, post-stenotic dilation [2], and a hooked appearance [14]. In our case, CECT did not reveal these findings, but instead demonstrated calcification at the orifice of the celiac axis, confirming that occlusion was due to atherosclerosis.

SS is an autoimmune disease characterized by keratoconjunctivitis sicca (dry eye) and xerostomia (dry mouth) due to lymphocyte-mediated destruction of exocrine glands, and it predominantly affects middle-aged women. Patients with SS and associated antibodies may also develop cardiovascular disease. Neonates exposed to maternal anti-SS A and anti-Ro/La autoantibodies can present with congenital heart block or atrioventricular (AV) block, as well as neonatal lupus [15]. In adults, however, these disorders are relatively rare because the mature AV node is believed to be more resistant to the damaging effects of anti-SSA antibodies [16]. Moreover, SS has been associated with atherosclerosis [17], aortic valve stenosis [18], and systemic aneurysms, as mentioned in the Introduction. Our patient had several atherosclerotic risk factors, including hypertension, hyperlipidemia, and obesity, and coexisting SS may have exacerbated vascular inflammation, contributing to celiac axis occlusion, PDAAs and CAs. Although a direct causal relationship cannot be established in this case, chronic immune-mediated vascular inflammation associated with SS may have predisposed this patient to systemic aneurysm formation. Corticosteroid therapy would also have been required. However, the successful staged multidisciplinary treatment of the aneurysms allowed the patient to remain free from corticosteroid therapy, thereby avoiding the risk of exacerbating MASH.

Regarding the therapeutic strategy, our case presented with both PDAAs and an AComA aneurysm at rupture risk grade IV. The three-year rupture risk of the AComA aneurysm was over 17% [19], necessitating urgent intervention for both lesions. Post-interventional management of CAs requires dual antiplatelet therapy (DAPT) with aspirin and clopidogrel for 3–6 months to prevent ischemic complications [20]. Performing CA treatment before PDAA bypass surgery would have exposed the patient to increased risks of stroke and bleeding during DAPT, potentially delaying aorto-hepatic bypass. Therefore, we prioritized the aorto-hepatic bypass and PDAA embolization, followed by aspirin and apixaban, which has a shorter half-life and a lower bleeding risk than other direct oral anticoagulants [21], thereby avoiding the risks associated with DAPT required for intracranial SAC.

Nine reports and 13 cases of combined bypass surgery and TAE for PDAAs, like our case, have been reported to date [4, 22–29] and the characteristic of these reports are summarized in Table 1. The surgical strategy for PDAAs caused by celiac axis stenosis or occlusion involves both securing blood flow to the celiac arterial system and treating the aneurysms [1]. In the cases of celiac axis stenosis or occlusion, hepatic and splenic perfusion is mainly supplied via PDAs and GDA. Revascularization of the celiac axis can be achieved by stenting or bypass surgery, including aorto-hepatic [22–24, 26, 27], aorto-splenic [4], renal-splenic [25], and aorto-celiac [26] bypasses. These procedures are followed by either simultaneous [24, 26, 27] or staged [4, 22, 23, 25, 26, 28, 29] TAE with coils or surgical removal [30] of the PDAAs. Lower-resistance bypasses tend to remain patent longer, with GSV grafts generally more durable than Dacron or expanded polytetrafluoroethylene grafts. Thus, the shortest possible GSV bypass is preferred. Although bypasses from the renal artery [25] or SMA [29] would have offered shorter graft courses in our case, we selected the abdominal aorta as the proximal anastomosis site to ensure stable inflow and reduce the risk of anastomotic stenosis. The graft was colored not to cause torsion and routed through the mesocolon near the ligament of Treitz and between the stomach and pancreas to provide the shortest course to the CHA. Furthermore, the aorto-hepatic bypass was also utilized as a coil embolization route.

In summary, we treated a rare case of PDAAs and CAs associated with SS. Our case highlights the potential for systemic aneurysm formation in patients with SS and demonstrates that staged multidisciplinary treatment can be safely and effectively performed.

**Table 1:** Reported cases of pancreaticoduodenal artery aneurysm treated with bypass surgery and trans-arterial embolization

Author	Publishedyear	Age/Sex	PDAAs type/diameter	Celiacaxis status/Etiology	Other aneurysms/diameter	Complicated AID	Treatment	Bypass	Graft	Interval between bypass and TAE	Follow-up(months)
Bageacu et al. [22]	2006	55/Unknown	Ruptured/15mm	Stenosis/MALS	None	None	TAE→bypass/Staged	Aorto-hepatic artery	Unknown	6 or 8 weeks	89
		43/Unknown	Ruptured/20mm	Stenosis/MALS	None	None	TAE→bypass/Staged	Aorto-hepatic artery	Unknown	6 or 8 weeks	78
		51/Unknown	Unruptured/18mm	Occlusion/MALS	None	None	Bypass→TAE/Staged	Aorto-hepatic artery	Unknown	Unknown	27
Teng et al. [23]	2006	46/M	Ruptured/19mm	Occlusion/Unknown	None	None	Bypass→TAE/Staged	Aorto-hepaticartery and SMA	Dacron	1 day	Unknown
Imamura et al. [24]	2011	61/M	Unruptured/20mm	Stenosis/MALS	Unknown	None	Bypass→TAE/Simultaneous	Aorto-hepatic artery	GSV	The same day	Unknown
Nakano et al. [25]	2014	47/M	Unruptured/35, 15mm	Occlusion/MALS	Unknown	None	Bypass→TAE/Staged	Renal-splenic artery	ePTFE	7 days	21
Simon et al. [26]	2017	39/F	Unruptured/40mm	Occlusion/Unknown	None	Psoriaticarthritis	TAE→bypass/Simultaneous	Aorto-hepatic artery	GSV	The same day	12
		61/F	Unruptured/20mm	Occlusion/Artherosclerosis	a CAA	Asthma	Bypass→TAE/Staged	Aorto-celiac artery	Dacron	1 month	18
Kii et al. [4]	2021	54/F	Unruptured/21,19, 8mm	Occlusion/MALS	None	None	Bypass→TAE/Staged	Aorto-splenic artery	Dacron	6 days	102
		39/M	Unruptured/23mm	Stenosis/MALS	Unknown	Unknown	Bypass→TAE/Staged	Aorto-splenic artery	Dacron	9 days	79
Tsujimoto et al. [27]	2021	62/F	Unruptured/10mm	Occlusion/MALS	None	None	Bypass→TAE/Simultaneous	Aorto-hepatic artery	ePTFE	The same day	12
Kubota et al. [28]	2022	Unknown	Unruptured/46, 9,11mm	Occlusion/Artherosclerosis	Unknown	None	Bypass→TAE/Staged	left CIA-splenic artery	Artificial vessel	7 days	≧11
Oshima et al. [29]	2024	74/F	Unruptured/22,15, 3, 20, 6mm	Occlusion/MALS	Unruptured SAA/12mm	Primary biliary cholangitis	Bypass→TAE/Staged	SMA-GDA	GSV	7-14 days	17
Current case	2026	69/F	Unruptured/12,14,12,8,9,8mm	Occlusion/Artherosclerosis	Unruptured CAAs/10, 3, 1.5mm	Sjögren's syndrome	Bypass→TAE/Staged	Aorto-hepatic artery	GSV	20 days	12

*PDAAs* Pancreaticoduodenal artery aneurysms, *AID* Autoimmune disease, *TAE* Trans-arterial embolization, *MALS* Median arcuate ligament syndrome, *SMA* Superior mesenteric artery, *GSV* Great saphenous veine, *PTFE* expanded Polytetrafluoroethylene, *CIA* Common iliac artery, *SAA* Splenic artery aneurysm, *GDA* Gastroduodenal artery, *CAAs* Cerebral artery aneurysms

## Data Availability

The dataset supporting the conclusions of this article is included within the article.

## Abbreviations

PDA: Pancreaticoduodenal Artery
PDAAs: Pancreaticoduodenal Artery Aneurysms
MAL: Median Arcuate Ligament
MALS: Median Arcuate Ligament Syndrome
SMA: Superior Mesenteric Artery
TAE: Trans-arterial Embolization
SS: Sjögren’s Syndrome
CAs: Cerebral Aneurysms
SSA: Sjögren’s Syndrome A
MASH: Metabolic Dysfunction–associated Steatohepatitis
CECT: Contrast-enhanced Computed Tomography
DPAAs: Dorsal Pancreatic Artery Aneurysms
ASPDAAs: Anterior Superior Pancreaticoduodenal Artery Aneurysms
AIPDAAs: Anterior Inferior Pancreaticoduodenal Artery Aneurysms
IPDAA: Inferior Pancreaticoduodenal Artery Aneurysm
A com A: Anterior Communicating Artery
MCA: Middle Cerebral Artery
GSV: Great Saphenous Vein
CHA: Common Hepatic Artery
GDA: Gastroduodenal Artery
PI: Pulsatility Index
ASPDA: Anterior Superior Pancreaticoduodenal Artery
DPAs: Dorsal Pancreatic Arteries
SAC: Stent-assisted Coil Embolization
AV: Atrioventricular
DAPT: Dual Antiplatelet Therapy
